# The Impact of Maternal Fructose Exposure on Angiogenic Activity of Endothelial Progenitor Cells and Blood Flow Recovery After Critical Limb Ischemia in Rat Offspring

**DOI:** 10.3390/ijms20102429

**Published:** 2019-05-16

**Authors:** Steve Leu, Kay L. H. Wu, Wei-Chia Lee, You-Lin Tain, Julie Y. H. Chan

**Affiliations:** 1 Institute for Translational Research in Biomedicine, Kaohsiung Chang Gung Memorial Hospital, Kaohsiung 833, Taiwan; wlh0701@yahoo.com.tw; 2 Department of Biotechnology, College of Life Science, Kaohsiung Medical University, Kaohsiung 833, Taiwan; 3 Department of Urology, Kaohsiung Chang Gung Memorial Hospital and Chang Gung University College of Medicine, Kaohsiung 833, Taiwan; dinor666@ms32.hinet.net; 4 Department of Pediatrics, Kaohsiung Chang Gung Memorial Hospital and Chang Gung University College of Medicine, Kaohsiung 833, Taiwan; tainyl@hotmail.com

**Keywords:** maternal fructose exposure, endothelial progenitor cell, critical limb ischemia

## Abstract

Adult metabolic syndrome is considered to be elicited by the developmental programming which is regulated by the prenatal environment. The maternal excess intake of fructose, a wildly used food additive, is found to be associated with developmental programing-associated cardiovascular diseases. To investigate the effect of maternal fructose exposure (MFE) on endothelial function and repair, which participate in the initiation and progress of cardiovascular disease, we applied a rat model with maternal fructose excess intake during gestational and lactational stage and examined the number and function of endothelial progenitor cells (EPCs) in 3-month-old male offspring with induction of critical limb ischemia (CLI). Results showed that the circulating levels of c-Kit+/CD31+ and Sca-1+/KDR+ EPC were reduced by MFE. In vitro angiogenesis analysis indicated the angiogenic activity of bone marrow-derived EPC, including tube formation and cellular migration, was reduced by MFE. Western blots further indicated the phosphorylated levels of ERK1/2, p38-MAPK, and JNK in circulating peripheral blood mononuclear cells were up-regulated by MFE. Fourteen days after CLI, the reduced blood flow recovery, lowered capillary density, and increased fibrotic area in quadriceps were observed in offspring with MFE. Moreover, the aortic endothelium-mediated vasorelaxant response in offspring was impaired by MFE. In conclusion, maternal fructose intake during gestational and lactational stage modulates the number and angiogenic activity of EPCs and results in poor blood flow recovery after ischemic injury.

## 1. Introduction

Metabolic disorder-associated cardiovascular risk factors, including diabetes mellitus, insulin resistance, dyslipidemia, and hypertension all cumulatively damage the endothelium [1,2]. On the other hand, endothelial dysfunction is also considered as the earliest abnormality in the development of vascular disease, and is linked to subsequent atherosclerosis progression and cardiovascular disease events [1,2]. In addition to high calorie/fat diet, recent studies have indicated that the change in dietary patterns of sugar intake, such as fructose, may be one of the causes of metabolic syndrome [3,4,5]. Fructose is wildly considered to play roles in regulating energy intake, body adiposity, and cardiovascular diseases [5,6]. In animal studies, insulin resistance, hypertension, and cardiac impairment have been found in rat fed with fructose diet [7,8,9], while clinical findings also indicated that intake of high quantities of fructose contributes to tissue insulin insensitivity, metabolic defects, and the development of a pre-diabetic state [10]. Of importance, fructose intake has been found to induce oxidative stress, inflammation, and dysfunction in endothelial cells [11,12,13], while the reduced population and impaired angiogenic activity of endothelial progenitor cells (EPCs) were also observed in rats fed with fructose [14]. EPC is a subset of bone marrow-derived cells, which participates in angiogenesis in the ischemic area [15]. In normal condition, EPCs reside in bone marrow, while only a few EPCs were found in the peripheral circulation. With ischemic stress, EPCs migrate from bone marrow to peripheral blood and then infiltrate into the ischemic area [16,17,18]. Both clinical and basic studies indicated that lower EPC counts and its impaired endothelial colony forming activity could be caused by metabolic disorders and have a higher incidence of cardiovascular events [19,20,21,22,23]. 

Recent studies have indicated that the prenatal environment and neonatal life exert a profound influence on the risk of disease in adulthood, particularly in the cardiovascular system [24,25,26]. Animal studies directly demonstrated that the maternal nutrient imbalance, such as nutrition restriction and overnutrition, results in hypertension, ventricular remodeling, and poor recovery from myocardial ischemia in offspring [26,27,28]. In endothelial cells, maternal chronic hypoxia or nutrient restriction during pregnancy impaired endothelial function in adult male rat offspring [29]. Another study further demonstrated that this maternal undernutrition-leaded endothelial dysfunction could not be revered by normal postnatal diet [30]. In our recent studies, we demonstrated that maternal high fructose intake during gestational and lactational stage leads to hypertension and renal developmental programming in offspring [31,32]. However, the effect of maternal fructose exposure on regulating the physiological function of endothelial cells and EPCs remains unknown. 

To clarify whether prenatal programming-induced by maternal fructose intake sets the level of vulnerability for endothelial dysfunction before birth and modulates the function and number of EPCs in adult offspring, we applied an experimental animal model to examine the effects of maternal fructose intake on the number and angiogenic activity of EPCs, as well as on the blood flow recovery after critical limb ischemia. 

## 2. Results

### 2.1. Maternal Fructose Exposure Impaired the Angiogenic Activity of Bone Marrow-Derived Endothelial Progenitor Cells

**Figure 1:**
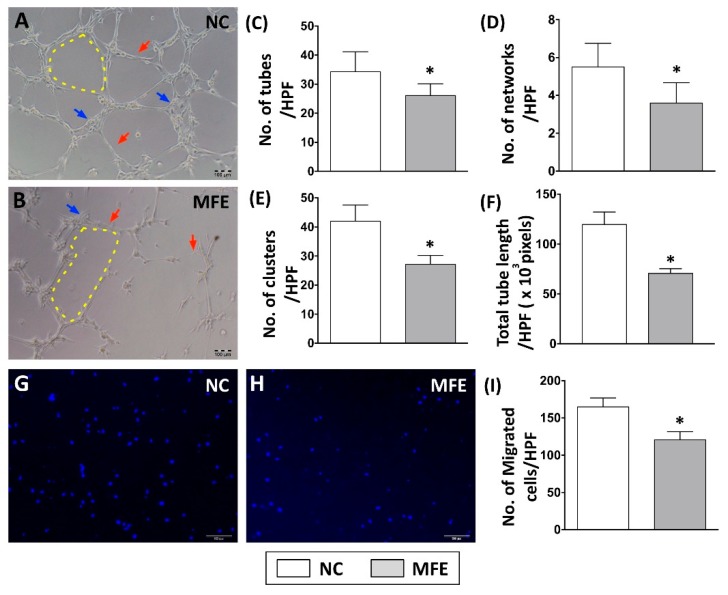
Maternal fructose exposure impaired angiogenic activity of bone marrow-derived endothelial progenitor cells. (**A**,**B**) In vivo angiogenic activity analysis (matrigel) on bone marrow-derived endothelial progenitor cells (BMDEPC) of 3-month-old offspring. (**C**) Calculation of tube formation in matrigel (red arrows). (**D**) Calculation of network formation in matrigel (yellow dotted line). (**E**) Calculation of cluster formation in matrigel (blue arrows). (**F**) Calculated of accumulated tube length. (**G**,**H**) Trans-well analysis to determine the migratory activity of BMDEPC. (**I**) Calculation of migrated cells in the trans-well assay. Results showed that the numbers of tube, network, and clusters in the MFE group were significantly lower than those in the NC group. NC, maternal control; MFE, fructose exposure. Scale bar in the lower-right corner indicates 100 μm. Error bars represent the standard deviation (SD). * Indicates statistical significance between NC and MFE group. *n* = 8 for each group.

The capacity of EPC differentiation into endothelial cells for angiogenesis is considered as one of the key factors in the blood flow recovery after ischemic injury. To examine whether maternal fructose exposure (MFE, maternal fructose intake during gestational, and lactational stage) participates in regulating endothelial differentiation and angiogenic activity of EPCs, bone marrow stromal cells from 3-month-old male offspring were isolated and cultured in endothelial growth medium for 14 days. Matrigel-based in vitro angiogenic activity assay and trans-well examination were performed to determine the capacity of bone marrow-derived endothelial progenitor cells (BMDEPCs) in angiogenesis. Results from the matrigel assay showed that the number of tubes, number of networks, number of clusters, and accumulated tube length of BMDEPCs were reduced by MFE (Figure 1A–F). Compared with normal offspring, the number of migrated cells shown in the trans-well assay was lower in offspring with MFE than that without MFE (Figure 1G–I). Both assays indicated that the angiogenic activity of BMDEPCs was impaired by maternal high fructose intake during the gestational and lactational stages.

### 2.2. Maternal Fructose Exposure Resulted in a Reduced Number of Circulating Endothelial Progenitor Cells in Offspring

**Figure 2:**
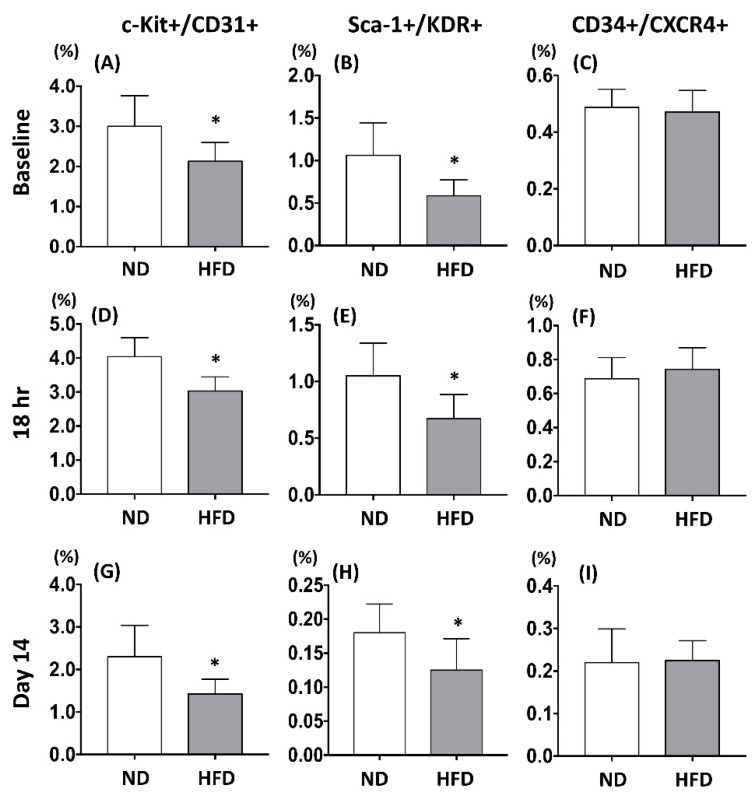
Maternal fructose exposure reduced the number of circulating endothelial progenitor cells. (**A**–**C**) The number of c-Kit+/CD31+, Sca-1+/KDR+, and CXCR4+/CD34+ in the peripheral bloods of the offspring prior to induction of critical limb ischemia (CLI). (**D**–**F**) The number of c-Kit/CD31, Sca-1/KDR, and CXCR4/CD34 in the peripheral bloods of the offspring 18 h post induction of CLI. (**G**–**I**) The number of c-Kit/CD31, Sca-1/KDR, and CXCR4/CD34 in the peripheral bloods of the offspring 14 days post induction of CLI. In normal condition, compared with the NC group, the number of circulating c-Kit+/CD31+ and Sca-1+/KDR+ EPCs were lower in the offspring with MFE. However, the number of CXCR4+/CD34+ cells was not affected by maternal fructose exposure. Eighteen hours and 14 days after induction of CLI. The circulating levels of c-Kit+/CD31+ and Sca-1+/KDR+ cells in MFE group was lower than that in the NC group, while levels of CXCR4+/CD34+ cells showed no difference between the two groups. NC, normal control; MFE, maternal fructose exposure; CLI, critical limb ischemia. Error bars represent the standard deviation (SD). * Indicates statistical significance between NC and MFE group. *n* = 8 for each group.

To examine the impact of MFE on regulating the population of circulating endothelial progenitor cells in adult offspring, peripheral mononuclear cells (PBMC) from 3-months-old male offspring were applied to flow cytometric examination with antibodies against EPC surface markers pairs (c-Kit/CD31, Sca-1/KDR, and CXCR4/CD34). Results showed that the population of EPCs with co-expression of c-Kit/CD31 or Sca-1/KDR was reduced in the offspring with maternal fructose exposure (Figure 2A,B). To determine the effect of MEF on modulating the number of circulating EPCs under ischemic stress, critical limb ischemia (CLI) was induced by ligation of the femoral artery in 3-months-old male offspring. Comparing with those without MFE, by 18 h and 14 days after CLI, the number of c-Kit/CD31 and Sca-1/KDR positively stained EPCs was lower in offspring with MFE (Figure 2D,E,G,H). However, the number of CXCR4+/CD34+ cells prior to CLI, 18 hrspost-CLI, and 14 days post-CLI showed no difference between two groups (Figure 2C,F,I). Those results indicated that the number and population of circulating EPCs in the adult offspring were altered by maternal fructose intake during the gestational and lactational stage.

### 2.3. Maternal Fructose Exposure Reduced the Blood Flow Recovery After Induction of Critical Limb Ischemia in Offspring 

**Figure 3:**
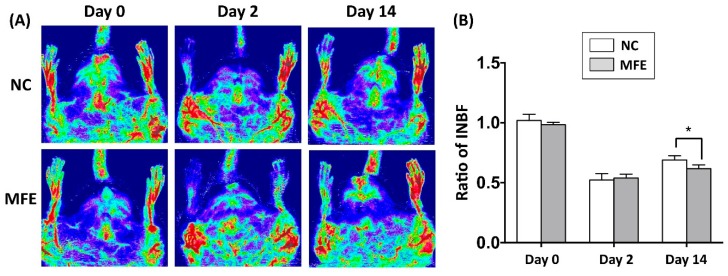
Maternal fructose exposure reduced blood flow recovery after induction of critical limb ischemia. (**A**) Laser Doppler scanning to determine the blood flow recovery after induction of CLI. (**B**) Quantitation and calculation of blood flow. The parameter ratio of ischemia to normal blood flow (INBF) was used to evaluate the blood flow recovery initiated by neovascularization after CLI. On day 2 after CLI and day 0 prior to CLI, the ratio of INBF showed no difference between NC and MFE groups. However, by day 14, the blood flow recovery in the NC group was significantly higher than the MFE group. NC, normal control; MFE, maternal fructose exposure; CLI, critical limb ischemia. Error bars represent the standard deviation (SD). * Indicates statistical significance between NC and MFE group. *n* = 8 for each group.

**Figure 4:**
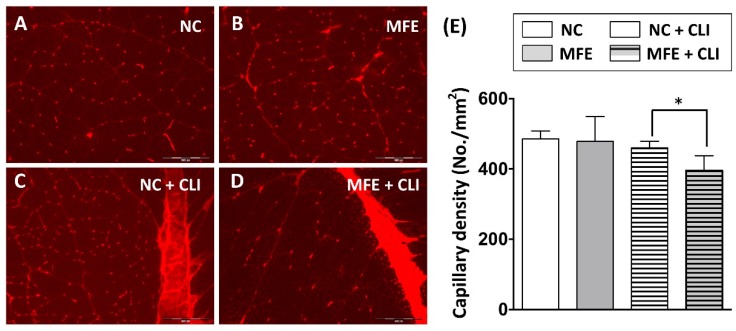
Capillary density in ischemic quadriceps was reduced by maternal fructose exposure. (**A**–**D**) Immunofluorescent staining to detect the distribution of CD31+ capillaries in quadriceps. (**E**) Calculation of capillary density in quadriceps. In normal condition, the capillary density showed no difference between the NC and MFE groups. By 14 days after induction of CLI, the capillary density in MFE group was significantly lower than that in NC group. NC, normal control; MFE, maternal fructose exposure; CLI, critical limb ischemia. Scale bar in the lower-right corner indicates 200 μm. Error bars represent the standard deviation (SD). * Indicates statistical significance between NC and MFE group. *n* = 8 for each group.

The number and migration of EPCs are considered as a pivotal step in neovascularization after ischemic injury. Following the flow cytometric analysis on circulating EPC, to determine the effect of MFE on blood flow recovery in adult offspring after ischemic injury, laser Doppler flowmetry was applied to detect for the blood flow in ischemic limbs of 3-months-old offspring (Figure 3A). By day 2 after induction of CLI and day 0 prior to CLI, the ratio of ischemia to normal blood flow (INBF) showed no difference between the two groups. By day 14 after CLI, the INBF in offspring with MFE was lower than that without MFE, indicating the blood flow recovery in offspring was impaired by MFE (Figure 3B). To determine the capillary density in the ischemic quadriceps, immunofluorescent staining with antibodies against CD31 were performed to detect the distribution of capillaries (Figure 4A–D). In normal conditions, MFE did not alter the capillary density in quadriceps (Figure 4E). By 14 days after CLI, the capillary density in ischemic quadriceps of offspring with MFE was lower than that without MFE. Taken together, results from blood flow measurement and capillary density examination all indicated that MFE not only reduced the number and angiogenic activity of EPCs in offspring but also impaired the neovascularization and blood flow recovery after ischemic injury in adult offspring. 

### 2.4. Maternal Fructose Exposure Increased Fibrosis in Quadriceps after Induction of Critical Limb Ischemia in Offspring

**Figure 5:**
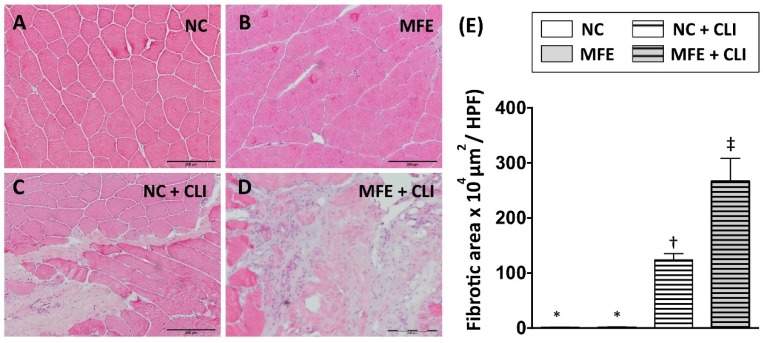
Maternal fructose exposure increased the fibrotic area in ischemic quadriceps after induction of critical limb ischemia. (**A**) Hematoxylin and eosin (H&E) staining for quadriceps. (**B**) Quantitation and calculation of the fibrotic area in the quadriceps. In normal conditions, no fibrotic area was found in quadriceps in both NC and MFE group. However, by 14 days after induction of CLI, the fibrotic area in MFE group was significantly higher than that in the NC group. NC, normal control; MFE, maternal fructose exposure; CLI, critical limb ischemia. Scale bar in the lower-right corner indicates 200 μm. Error bars represent the standard deviation (SD). Groups with different symbols (*, †, ‡), *p*< 0.05. *n* = 8 for each group.

Following measurement of blood flow recovery, histopathological examination with hematoxylin and eosin (H&E) staining was performed to examine the effects of MFE on fibrosis after induction of CLI in adult offspring (Figure 5A–D). In normal conditions, there was no fibrosis observed in quadriceps of offspring with or without MFE. By 14 days after induction of CLI, tissue fibrosis in ischemic quadriceps was observed in both two groups. Along with the reduced capillary density and poor blood flow recovery in ischemic limbs, MFE also increased the fibrotic area in the ischemic quadriceps of adult offspring (Figure 5E). 

### 2.5. Intra-Cellular Signal Transduction in Peripheral Blood Mononuclear Cells was Regulated by Maternal Fructose Exposure

**Figure 6:**
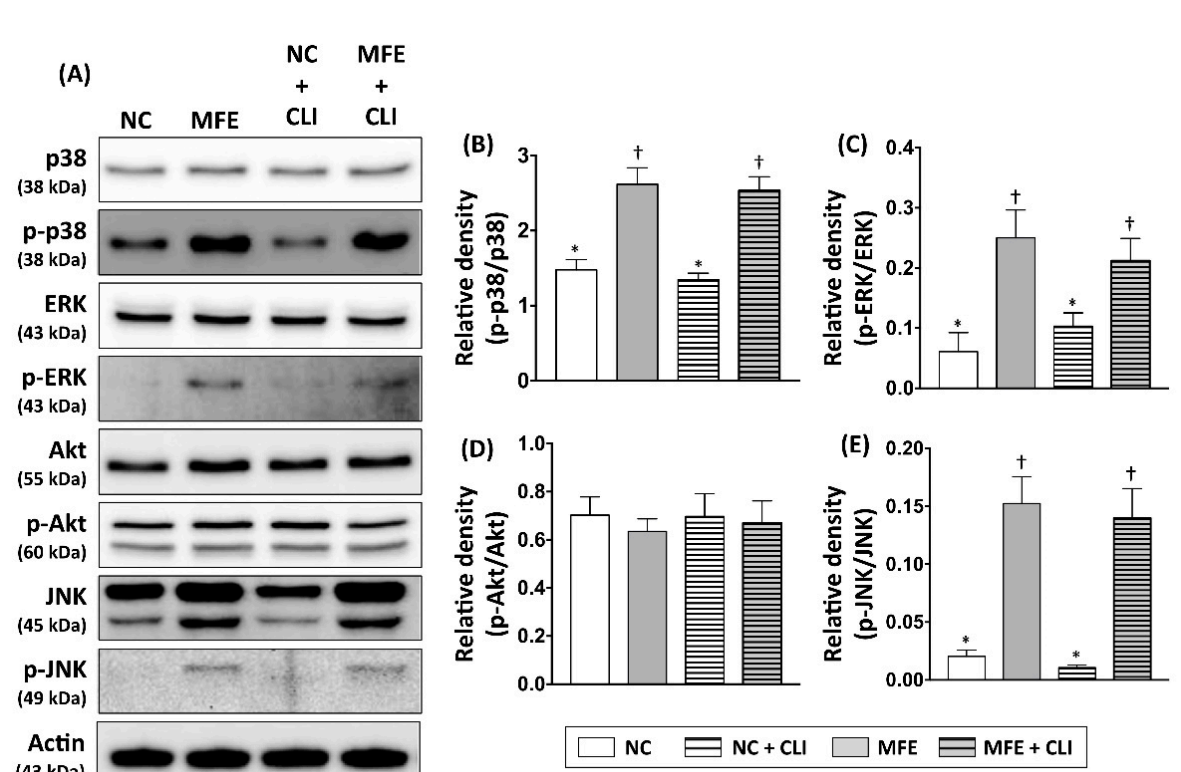
Maternal fructose exposure regulated expression and phosphorylation levels of angiogenesis-associated signaling in bone marrow cells. (**A**) Western blots to detect the expression and phosphorylation levels of several intracellular signaling proteins, including Akt, ERK1/2, p38-MAPK, and JNK in peripheral mononuclear cells (PBMC) of offspring 14 days post induction of CLI. (**B**) Quantitative data represent the ratio of phosphorylated p38-MAPK expression to total p38-MAPK expression. (**C**) Quantitative data represent the ratio of phosphorylated ERK1/2 expression to total ERK1/2 expression. (**D**) Quantitative data represent the ratio of phosphorylated Akt expression to total Akt expression. (**E**) Quantitative data represent the ratio of phosphorylated JNK expression to total JNK expression. NC, normal control; MFE, maternal fructose exposure; CLI, critical limb ischemia. Error bars represent the standard deviation (SD). Groups with different symbols (*, †), *p*< 0.05. *n* = 8 for each group.

To examine whether intracellular signaling that participates in cellular proliferation, stress response, and endothelial differentiation in circulating EPCs of offspring were regulated by MFE, Western blots were performed to detect the expression and activation levels of Akt, ERK, p38-MAPK, and JNK in peripheral blood mononuclear cells of adult offspring (Figure 6A). By 14 days post induction of CLI, both expression and phosphorylation levels of Akt showed no difference among groups (Figure 6D). Although the expression levels of p38-MAPK and ERK were not regulated by either MFE or CLI, the phosphorylated levels of those proteins were significantly increased with MFE (Figure 6B,C). In addition, both expression and phosphorylation levels of JNK in the MFE group were higher than that in the NC group (Figure 6E). However, the induction of CLI showed no significant synergistic effects on regulating expression and phosphorylation of p38-MAPK, ERK, and JNK. 

### 2.6. Maternal Fructose Exposure Reduced the Endothelium-Mediated Vasorelaxant Response in Offspring

**Figure 7:**
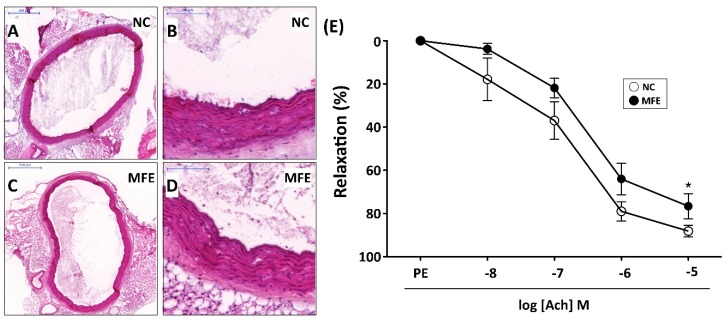
Maternal fructose exposure reduced the endothelium-mediated vasorelaxant response. (**A**,**B**) Hematoxylin & Eosin (H&E) staining for a cross-section of the aorta in NC offspring. (**C**,**D**) H&E staining for a cross-section of aorta in MFE offspring. (**E**) Endothelium-mediated relaxation to acetylcholine was assessed in aortic rings from 3-month-old offspring. Data were calculated as changes from the contraction induced by phenylephrine. NC, normal control; MFE, maternal fructose exposure; Ach, acetylcholine. Scale bar in the upper-left corner of (**A**) and (**C**) indicates 500 μm. Scale bar in the upper-left corner of (**B**) and (**D**) indicates 100 μm. Error bars represent the standard deviation (SD). * Indicates statistical significance between NC and MFE group. *n* = 8 for each group.

In addition to neovascularization, the inefficient number and angiogenic activity of circulating EPC is also associated with vascular remodeling and functional impairment. Following the examination on circulating EPCs, aortas from adult offspring were also isolated for histopathological and vasorelaxant examination to determine the effect of MFE on vascular remodeling. Although results from H&E staining indicated that there was no significant difference in the integrity of intima and the thickness of media between two groups (Figure 7A–D), the effect of MFE on impairing endothelium-mediated vasorelaxation was observed. Compared to normal offspring, the aortic vasorelaxant response to acetylcholine in offspring with MFE was lower than that without MFE (Figure 7E), indicating the endothelial function, particularly in regulating vasorelaxation, was modulating by MFE. 

## 3. Discussion

In the present study, a rat model was applied to examine the effect of excessive fructose intake during the gestational and lactational stages on regulating differentiation and angiogenic activity of EPCs in adult offspring. The CLI rat model was also utilized to examine the effect of MFE on blood flow recovery and neovascularization in ischemic limbs of adult offspring. Results showed that not only the circulating population but also the angiogenic activity of bone marrow-derived EPCs in 3-month-old offspring were down-regulated by maternal fructose exposure (Figure 1 and Figure 2). Furthermore, the blood flow recovery (Figure 3) and capillary density (Figure 4) in quadriceps by 14-day after CLI induction were lower in the offspring with MFE. Along with the decreasing in blood flow recovery, the increased tissue fibrosis was observed in ischemic quadriceps of offspring with MFE (Figure 5). Results from Western blotting further indicated the activation of several intracellular signal transductions, including p38MAPK, EKR, and JNK in peripheral mononuclear cells were up-regulated by MFE (Figure 6). Finally, although no significantly morphological alternation was observed, the reduced endothelium-mediated vasorelaxant response was found in the offspring with MFE (Figure 7). 

Accumulating evidence from animal and clinical studies suggested that the effect of maternal environmental insults, including nutrition imbalance, make effects on organogenesis and physiological function maintained in offspring [33,34]. Not only metabolic syndromes, but recent studies also indicated that maternal nutrition insults affect the fetal immune system development and impair the learning and memory in offspring [35,36]. It is reasonable that maternal nutrition insults make systemic effects on developmental programming in the offspring, including the wildly distributed endothelial cells. Previous studies in cultured cell and animal models have indicated that excess fructose intake results in endothelial functional impairment [13,37,38]. In this study, we further demonstrated that the aortic endothelium-mediated vasorelaxant response in adult offspring was impaired by maternal excessive fructose intake (Figure 7). The finding on aortic relaxation echoed our recent study in which the programmed hypertension was observed in adult offspring with MFE [39]. In patients with coronary artery disease, the lower number of circulating EPC was found to be correlated with endothelial dysfunction assessed by coronary angiography during intracoronary acetylcholine infusion and flow-mediated vasodilatation [20,40]. In the present study, both lower numbers of circulating EPCs and impaired aortic endothelium-mediated vasorelaxant responses were observed in offspring with maternal fructose excessive intake. However, further studies are needed to clarify whether the endothelial dysfunction in offspring is caused by maternal fructose exposure-induced decrease of circulating EPCs. In addition to the number of EPCs, the impaired angiogenic activity of bone marrow-derived EPCs was also observed in offspring with maternal fructose exposure. Like the number of EPC, the in vitro angiogenic activity of EPC is also considered to be associated with metabolic syndrome and diabetes [41]. An animal study also demonstrated that the intake of fructose impaired angiogenic activity of EPCs and aggravated cerebral ischemic injury [14]. In this study, we further indicated that maternal fructose exposure results in impaired EPC function and poor injury recovery after induction of limb ischemic in offspring. 

Several intracellular signal transductions, including Akt and MAPKs, have been found to participate in regulating angiogenic activity, proliferation, and stress response of EPCs and endothelial cells [42,43,44]. In the present study, the phosphorylation levels of MAPK-p38, ERK, and JNK were up-regulated by MFE, while the phosphorylation level of Akt was not affected. In EPCs treated with Doxorubicin, the elevation of phosphorylated MAPK-p38 was observed along with senescence phenotype [44]. Another study also indicated that inhibition of MAPK-p38 increases the proliferation of EPCs [43] as well as counteract senescence in corneal endothelial cells [45]. Of interest, the upregulated phosphorylation of JNK, which reported as an antagonistic role against MAPK-p38 in cellular senescence [44], was also observed in circulating EPCs of adult offspring with MFE. The activation of ERK, another MAPK signaling protein, was also reported to suppress pluripotent gene in embryonic stem cells [46] and participate in angiotensin II-induced senescence in endothelial cells [46]. Taken together, it is reasonable that the maternal fructose exposure-induced functional impairment and number reduction of EPC may be through the regulation on MAPK-p38 and ERK signaling. To determine the relationship between fructose intake and MAPK signaling, a previous study has indicated that the excessive fructose intake would increase the uric acid level and lead to MAPK signaling activation and endothelial injury [12], while another study also demonstrated that maternal fructose intake during pregnancy drives placental uric acid production and increase fetal serum level of fructose, glucose, and triglyceride [47]. In this study, we further indicated that MFE regulated and MAPK signaling in circulating EPCs and lead to endothelial dysfunction, lesser revascularization, and poor injury recovery after CLI. However, whether uric acid or other fructose-derived metabolites participate in the developmental programming of EPC and endothelial cells in offspring remains unknown and needs to be further investigated. In addition to the regulation on signal transduction, increasing evidence also suggests that the epigenetic regulation of gene expression, such as DNA methylation and histone acetylation, also plays a crucial role in the developmental programming of metabolic syndrome [48,49]. It is worth to consider that epigenetic regulation may participate in MFE-induced developmental programming of endothelial cells and EPCs. 

In conclusion, maternal fructose intake during gestational and lactational stage modulates the number and angiogenic activity of EPCs in the adult offspring. In addition, the reduction in endothelium-mediated vasorelaxant response, poor revascularization, and blood flow recovery, as well as increased tissue fibrosis were found in CLI offspring with MFE. 

## 4. Materials and Methods 

### 4.1. Ethics 

All experimental animal procedures were approved by the Institute of Animal Care and Use Committee at Kaohsiung Chang Gung Memorial Hospital (No. 2014093003, approved on 23 October 2014) and performed in accordance with the Guide for the Care and Use of Laboratory Animals (NIH publication No. 85-23, National Academy Press, Washington, DC, USA, revised 1996).

### 4.2. Animals and Induction of Critical Limb Ischemia in Rats

All animal experiments in this study were housed in an Association for Assessment and Accreditation of Laboratory Animal Care International (AAALAC)-certified animal facility in our hospital with controlled temperature and light cycles (24 °C and 12/12 light cycle). For maternal high fructose diet feeding, a laboratory rat chow with 60% fructose (Harlan Laboratories, Hayward, CA, USA) was provided to the 10-week-old female mother Sprague Dawley (SD) rats immediately following the mating period and at the labor day until weaning on postnatal day 21, at which time male offspring were separated into groups by litter and fed with standard chow. For induction of critical limb ischemia, 3-month-old male rats were anesthetized by inhalation of 2% isoflurane and then placed in a supine position on a warming pad at 37 °C with left hind limbs shaving. Under sterile conditions, the left femoral artery, small arterioles, and circumferential femoral artery were exposed and ligated over their proximal and distal portions, followed by removing. To avoid the presence of collateral circulation, the branches were removed together. However, the veins were left intact during the procedure. 

### 4.3. Isolation and Culturing of Bone Marrow-Derived Endothelial Progenitor Cell 

Bone marrow stromal cells were discovered from femur bones of 3-month-old offspring and resuspended in 10 mL saline and then centrifuged again at 600× *g* for 5 min at room temperature. After resuspension in 5 mL phosphate buffered saline (PBS), cells were filtered through a 40 μm filter. Cells then were centrifuged at 600× *g* for 5 min at room temperature and resuspended in endothelial cell growth medium (EGM-2, Lonza, Basel, Switzerland). Cells were then cultured in EGM-2 for 14 days to induce endothelial differentiation. After culturing, flow cytometric analysis was performed to detect the surface markers of endothelial progenitor cells (EPCs).

### 4.4. Flow Cytometric Quantification of Endothelial Progenitor Cells. 

For blood sampling at different time points (baseline, and at 18 h or on days 14 after induction of CLI), blood sampling from tail vein was performed using a 25# needle. After treatment with red blood cell-lysing buffer, the cells remained were labeled with appropriate antibodies. Flow cytometric analysis for identification of cell surface markers was performed based on our previous reports [50]. Briefly, the cells were incubated for 30 min with primary antibodies, including PE-conjugated antibodies (against CD34, Sca-1, CD31, BD Biosciences, San Jose, CA, USA), FITC-conjugated antibody against c-Kit (BD Biosciences), anti-CXCR4 (Abcam, Cambridge, MA, USA), and anti-KDR (NeoMarkers, Fremont, CA, USA) antibodies which were further recognized by Alexa flour 488-conjugated secondary antibodies (Invitrogen, Carlsbad, CA, USA). Isotype-identical antibodies (IgG) served as controls. Flow cytometric analyses were performed by utilizing a fluorescence-activated cell sorter (Beckman Coulter FC500 flow cytometer, Brea, CA, USA).

### 4.5. Measurement of Blood Flow with Laser Doppler 

Rats were anesthetized by inhalation of 2% isoflurane prior to CLI induction and at days 2 and 14 after CLI induction prior to being sacrificed. The rats were placed in a supine position on a warming pad at 37 °C and the blood flow was detected in both inguinal areas by a Laser Doppler scanner (moorLDLS, Moor, Co., Axminster, UK) The ratio of flow in the left (ischemic) leg and right (normal) leg was computed. The rats were sacrificed and the quadriceps muscle was collected for histopathological and biochemical examination.

### 4.6. Transwell Migratory Assay and Matrigel Angiogenesis Assay

Trans-well membranes (5 μm; Costar, St. Louis, MO, USA) were coated on both sides with fibronectin (2.5 μg/mL; Roche, Mannheim, Germany) overnight at 4 °C. 5 × 10^4^ cells were resuspended in M199 medium with 0.5% FBS and loaded into the upper chamber. The assembled trans-well system was then incubated at 37 °C in 5% CO_2_ for 18 h. After incubation, cells that were remaining on the upper surface of the trans-well membranes were mechanically removed and cells that had migrated to the lower surface were fixed with 4% formaldehyde. For quantification of cell nuclei, the migrated cells were stained with DAPI. Cells migrating into the lower chamber were observed with a fluorescence microscope (BX-51, Olympus, Tokyo, Japan) and counted in 5 random fields using the software Image-Pro Plus (Media Cybernetics, Bethesda, MD, USA). For tube formation assay in matrigel, 96-well microplates (Nunc, Waltham, MA, USA) were coated with matrigel (40 mL/well) (BD, Piscataway, NJ, USA) and allowed to polymerize at 37 °C for 30 min. 3 × 10^4^ bone marrow-derived EPCs were added into each well and covered with matrigel mixture with EGM-2. Tube formation was recorded 6 h post cell seeding and by IX51 inverted microscope (Olympus, Tokyo, Japan). 

### 4.7. Western Blot

Equal amounts (10–30 μg) of protein extracts from peripheral blood mononuclear cells were loaded and separated by SDS-PAGE using 7.5–12% acrylamide gradients. Following electrophoresis, the separated proteins were transferred electrophoretically to a polyvinylidene difluoride (PVDF) membrane (Amersham Biosciences, Buckinghamshire, UK). Nonspecific proteins were blocked by incubating the membrane with a blocking solution (5% nonfat dry milk in T-TBS containing 0.05% Tween 20) for 30 min. The membranes were incubated with the primary antibodies phosphorylated-Akt (1:1000, Cell Signaling, Danvers, MA, USA), Akt (1:1000, Cell Signaling), phosphorylated-ERK (1:1000, Calbiochem, St. Louis, MO), ERK (1:1000, Calbiochem), phosphorylated-JNK (1:1000, Abcam, Cambridge, MA, USA), JNK (1:1000, Abcam), phosphorylated p38-MAPK (1:1000, Sigma, St. Louis, MO, USA), p38-MAPK (1:5000, Abcam), and β-actin (1:1000, Cell Signaling) for 1 h at room temperature. Signals were detected with HRP-conjugated goat anti-mouse or goat anti-rabbit with ECL (Perkin Elmer, Waltham, MA, USA).

### 4.8. Histopathological and Immunostaining 

For immunofluorescent staining, isolated quadriceps were mounted in OCT and used for preparing cryosections. Sections were fixed and permeated with ice-cold acetone or 4% paraformaldehyde with 0.5% Triton X-100, and then incubated with antibodies against CD31 at 4 °C overnight. Coverslips or slides were then incubated with Alex594-conjugated goat anti-mouse or rabbit IgG (Invitrogen, Carlsbad, CA, USA). After counterstaining with DAPI, sections were examined under a fluorescent microscope.

### 4.9. Measurement of Endothelium-Mediated Vasorelaxant Response

The descending aorta was isolated, cleaned, and cut into slices 2 mm in length for evaluating the contractile and relaxant response as previously reported [51] with modifications. Briefly, aortic rings were carefully mounted on an isometric force transducer (XDFT05; Singa, Taiwan) with a tension of 2.0 g and placed in an organ chamber filled with Krebs solution (NaCl, 99.01 mM; KCl, 4.69 mM; CaCl_2_, 1.87 mM; MgSO_4_, 1.20 mM; K_2_HPO_4_, 1.03 mM; glucose, 11.1 mM) maintained at pH 7.4 and bubbled with 95% O_2_–5% CO_2_. After equilibration of 40 min, 1 mM of phenylephrine (PE) was added to the organ chamber for the assessment of contractile activity and then 30 mM of acetylcholine was added to assess the endothelial integrity. After washing and re-equilibration for 20 min, the cumulative concentration of acetylcholine (Ach) was added in the organ chamber followed by 1 mM of PE to evaluate the endothelium-dependent vasorelaxant response. All data were acquired and analyzed using Panlab Compact Organ Baths (Harvard Bioscience, Holliston, MA, USA)

### 4.10. Statistical Analysis 

Data were expressed as mean values (mean ± SD). The significance of differences between the two groups was evaluated with *t*-test. The significance of differences among the groups was evaluated using a one-way ANOVA, followed by a Bonferroni multiple comparison post-hoc test. Statistical analyses were performed using Prism 6 statistical software (GraphPad Software, La Jolla, CA, USA). A probability value <0.05 was considered statistically significant.

## Abbreviations

MFE	Maternal fructose exposure
EPC	Endothelial progenitor cell
CLI	Critical limb ischemia
PBMC	Peripheral blood mononuclear cell
